# Early production of IL‐17A by γδ T cells in the trachea promotes viral clearance during influenza infection in mice

**DOI:** 10.1002/eji.201948157

**Published:** 2019-12-04

**Authors:** Miguel Palomino‐Segura, Irene Latino, Yagmur Farsakoglu, Santiago F. Gonzalez

**Affiliations:** ^1^ Institute for Research in Biomedicine Università della Svizzera italiana via Vincenzo Vela 6 Bellinzona Switzerland; ^2^ Graduate School of Cellular and Molecular Sciences Faculty of Medicine University of Bern Bern Switzerland; ^3^ Salk Institute for Biological Studies La Jolla CA USA

**Keywords:** γδ T cell, IL‐17A, trachea, influenza virus, neutrophils

## Abstract

The innate immune response generated against influenza infection is critical for the inhibition of viral dissemination. The trachea contains different types of innate immune cells that protect the respiratory tract from pathogen invasion. Among them, γδ T cells have the ability to rapidly generate large amounts of pro‐inflammatory cytokines to preserve mucosal barrier homeostasis during infection. However, little is known about their role during the early phase of influenza infection in the airways. In this study, we found that, early after infection, γδ T cells are recruited and activated in the trachea and outnumber αβ T cells during the course of the influenza infection that follows. We also showed that the majority of the recruited γδ T cells express the Vγ4 TCR chain and infiltrate in a process that involves the chemokine receptor CXCR3. In addition, we demonstrated that γδ T cells promote the recruitment of protective neutrophils and NK cells to the tracheal mucosa. Altogether, our results highlight the importance of the immune responses mediated by γδ T cells.

## Graphical abstract

Innate recognition of influenza virus infection is critical for the control of the viral dissemination. Among the innate immune cells recruited to the infected trachea, Vγ4 + γδ T cells rapidly secrete IL‐17A that contributes to neutrophil recruitment and the elimination of virus‐infected cells from the trachea.

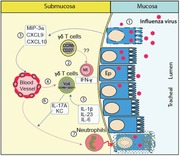

## Introduction

The trachea plays a critical role in the host defense against influenza virus by limiting viral replication and supporting the development of the adaptive immune responses during the early phase of viral infection 1, 2. The tracheal mucosa is characterized by a high frequency of innate immune cells that respond to external threats 2. Among them, γδ T cells are characterized by a diverse range of TCRs, with specific Vγ‐Vδ combinations, which are used as indicators of their anatomical location and function 3. Regarding their protective role, resident γδ T cells colonize from birth the epithelial layers of mucosal tissues where they are able to respond quickly to injury 4 or infections 5 to preserve homeostasis. Moreover, γδ T cells protect against pathogen invasion by combining characteristics typical of the adaptive immune system with rapid innate‐like responses 5.

The importance of γδ T cell functions has been frequently associated with the immune response against bacteria 6 and parasites 7. However, a growing number of studies have demonstrated a protective role of γδ T cells against viruses 8, 9, 10, 11. During influenza infection, γδ T cells have been shown to exert protective roles in the recovery phase after infection 12 or during secondary challenges with a different influenza A virus strain 13. In addition, human Vγ9Vδ2 T cells have been shown to directly eliminate virus‐infected cells in vitro 14 and in humanized mice 15.

One of the main characteristics of the γδ T cell response is their ability to produce large amounts of pro‐inflammatory cytokines, such as IFN‐γ and IL‐17A, without the need of TCR engagement 16. IFN‐γ is a cytokine that plays a key role in the host's antiviral defense and can be produced by various types of innate immune cells at early time points after infection 17. IL‐17A, on the other hand, is an important mediator in mucosal immunity and promotes the accumulation of inflammatory cells 18.

While the role of IFN‐γ in the context of influenza or viral infection is well documented 17, 19, the relevance of IL‐17A in innate immunity has been mainly studied in the context of lung bacterial infections 20, 21. In addition, IL‐17A has been recently shown to be an important immune modulator during viral infections 22. Furthermore, previous studies have demonstrated that γδ T cells are the main source of IL‐17A in lungs during influenza infection 23, 24. However, the precise role of IL‐17‐producing γδ T cells (γδ17 T cells) during the early response to influenza virus remains unknown.

In this study, we found that a subset of γδ T cells expressing typical receptors of γδ17 T cells was recruited to the tracheal epithelium early after infection. In addition, these γδ T cells produced large amounts of IL‐17A but low levels of IFN‐γ. We also discovered that the presence of γδ T cells promoted the recruitment of neutrophils and NK cells and was essential for the control of viral replication after influenza infection.

## Results

### Influenza infection induces the recruitment of activated γδ T cells to the trachea

**Figure 1:**
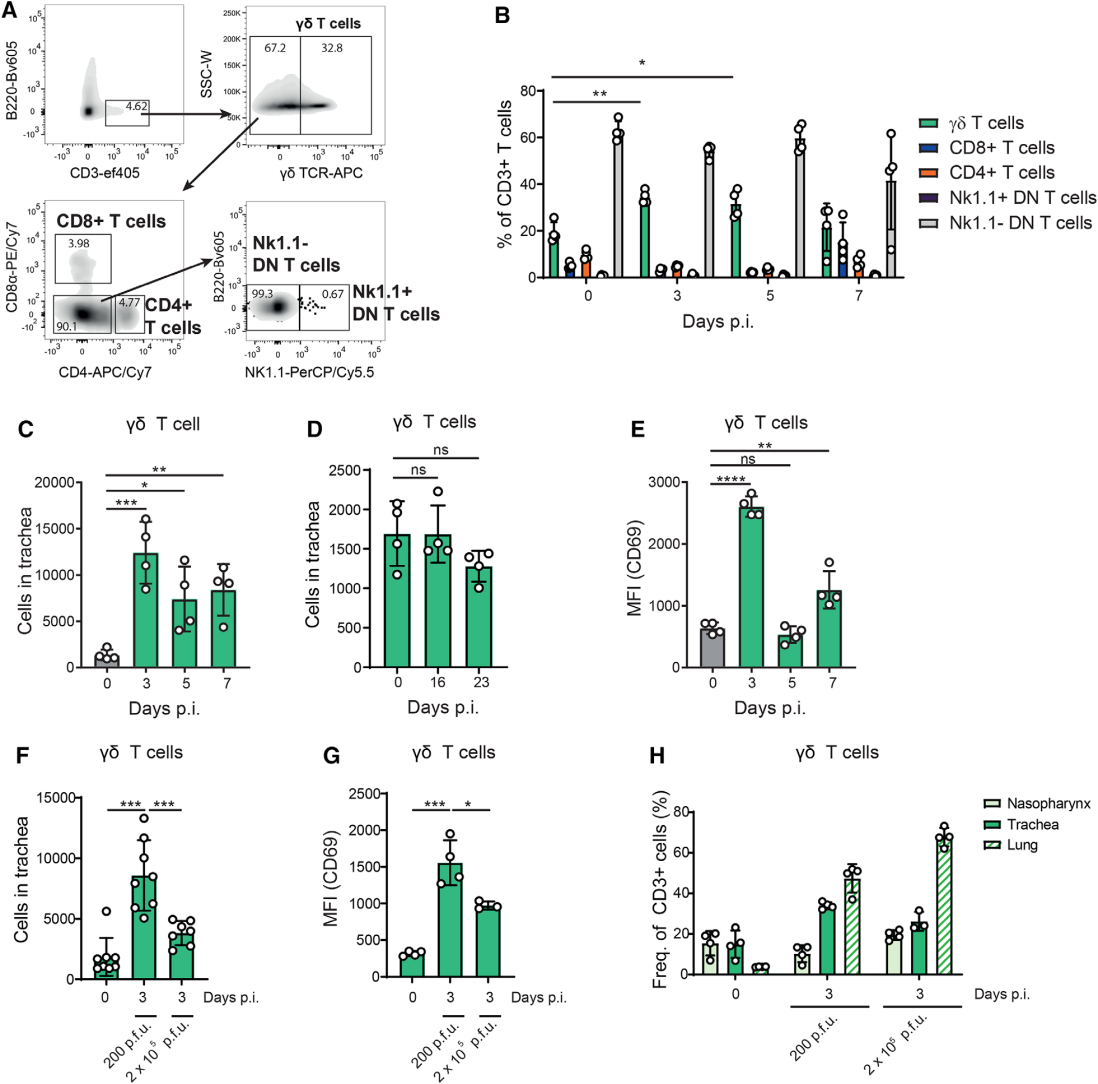
γδ T cell dynamics in trachea from mice infected with influenza virus. (A) Representative flow cytometric characterization of γδ T cells (CD3^+^/B220^–^/γδ TCR^+^), CD8^+^ T cells (CD3^+^/B220^–^/γδ TCR^–^/CD8α^+^/CD4^–^), CD4^+^ T cells (CD3^+^/B220^–^/γδ TCR^–^/CD8α^–^/CD4^+^), NK1.1^+^ double negative (DN) T cells (CD3^+^/B220^–^/γδ TCR^–^/CD8α^–^/CD4^–^/NK1.1^+^), and NK1.1^–^ DN T cells (CD3^+^/B220^–^/γδ TCR^–^/CD8α^–^/CD4^–^/NK1.1^–^) in mouse trachea. (B) Frequency of T cell subsets in trachea at 0, 3, 5, and 7 d.p.i. with PR8 (*n* = 4 mice/group). (C) Flow cytometry quantification of total numbers of γδ T cells in trachea at 0, 3, 5, and 7 d.p.i. (*n* = 4 mice/group). (D) Flow cytometry quantification of total numbers of γδ T cells in trachea at 0, 16, and 23 d.p.i. (*n* = 4 mice/group). (E) MFI expression levels of CD69 in tracheal γδ T cells at 0, 3, 5, and 7 d.p.i. (*n* = 4 mice/group). (F) Flow cytometry quantification of total numbers of γδ T cells in trachea at 0 and 3 d.p.i. with 200 or 2 × 10^5^ PFUs of PR8 (*n* = 7–8 mice/group). (G) MFI expression levels of CD69 in tracheal γδ T cells at 0 and 3 d.p.i. with 200 or 2 × 10^5^ PFUs of PR8 (*n* = 4 mice/group). (H) Flow cytometric analysis showing the frequency of γδ T cell in nasopharynx, trachea and lungs at 0 and 3 d.p.i. with 200 and 2 × 10^5^ PFUs of PR8 (*n* = 4 mice/group). The presented data are representative of at least three independent experiments (A, B, C, and E) or two independent experiments (D, F, G, and H) and analyzed using flow cytometry. Results are given as mean ± SD. Statistical significance was determined by Two‐tailed Student's *t*‐test (B, C, E) or Mann–Whitney *U*‐test (D, F, G). ns, *p* > 0.05; ^*^ *p* < 0.05; ^**^ *p* < 0.01; ^***^ *p* < 0.001; ^****^ *p* < 0.0001.

To study the involvement of different T cell subsets in the initial response against influenza, we characterized by flow cytometry the changes in cell populations on the basis of their expression of different surface markers: γδ T cells (CD3^+^/B220^–^/γδ TCR^+^), CD8^+^ T cells (CD3^+^/B220^–^/γδ TCR^–^/CD8α^+^/CD4^–^), CD4^+^ T cells (CD3^+^/B220^–^/γδ TCR^–^/CD8α^–^/CD4^+^), NK1.1^+^ double negative (DN) T cells (CD3^+^/B220^–^/γδ TCR^–^/CD8α^–^/CD4^–^/NK1.1^+^), and NK1.1^–^ DN T cells (CD3^+^/ B220^–^/γδ TCR^–^/CD8α^–^/CD4^–^/NK1.1^–^; Fig. 1A). Then, we assessed the frequency of the different subtypes of T cells in uninfected controls and during the first 7 days postinfection (d.p.i.) with a low dose of influenza virus (200 PFUs/animal) strain A/Puerto Rico/8/1934 H1N1 (PR8). We observed that the frequency of γδ T cells significantly increased at 3 and 5 d.p.i. with respect to uninfected controls (Fig. 1B). Regarding total numbers of γδ T cells in the trachea, we observed a similar trend, having the highest number at 3 d.p.i. (1C), and returning to basal levels at 2 weeks postinfection (Fig. 1D). Next, we evaluated the activation of γδ T cells by examining the expression of CD69 during the first 7 d.p.i. We observed that γδ T cells were highly activated at 3 d.p.i. (Fig. 1E). Finally, we assessed how γδ T cells numbers and activation levels change when animals are infected with a higher dose of influenza virus (2 × 10^5^ PFUs/mice) that generates an acute pulmonary infection. We observed that the numbers of γδ T cells number and their activation in trachea were reduced (Fig. 1F and G, respectively) and that γδ T cells frequency increased in the lungs (Fig. 1H).

### Most γδ T cells present in trachea express the Vγ4 TCR chain

**Figure 2:**
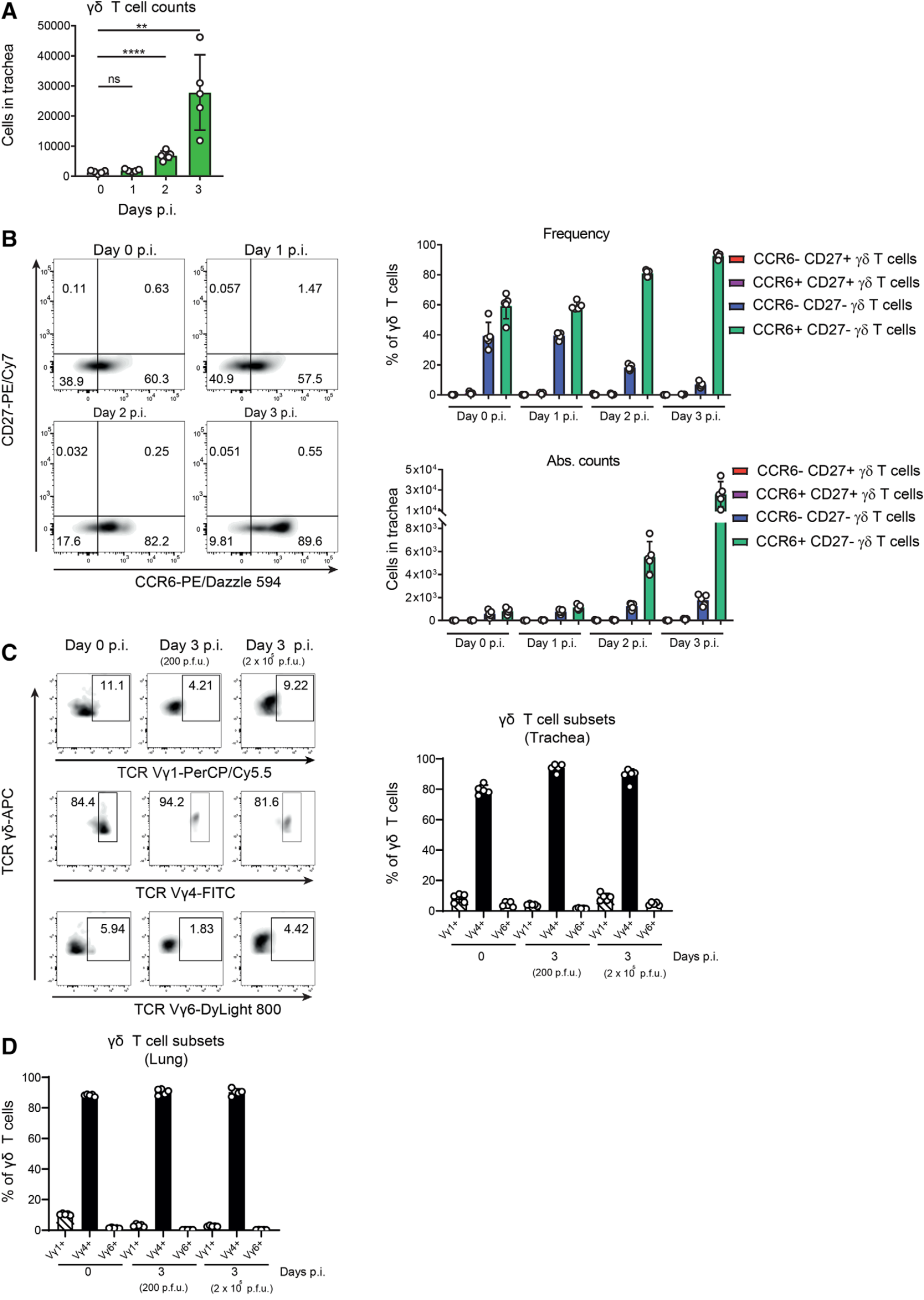
Most γδ T cells present in trachea express the Vγ4 TCR chain. (A) Flow cytometric quantification of total numbers of γδ T cells in trachea at 0, 1, 2, and 3 d.p.i. (*n* = 5 mice/group). (B) (Left panel) Representative scatterplots showing the characterization of the different γδ T cell subtypes by flow cytometry according to the surface expression of CCR6 and CD27 in trachea at 0, 1, 2, and 3 d.p.i. (Right) Frequency (top) and total numbers (bottom) of the different γδ T cell subtypes at 0, 1, 2, and 3 d.p.i. (*n* = 5 mice/group). (C) Representative scatterplots showing the characterization of the different γδ T cell subtypes by flow cytometry according to the expression of their Vγ chains in trachea at 0 and 3 d.p.i. (Right) Flow cytometric quantification of frequency of the different γδ T cell subtypes in trachea at 0 and 3 d.p.i. with 200 or 2 × 10^5^ PFUs of PR8 (*n* = 5 mice/group). (D) Flow cytometric quantification of frequency of the different γδ T cell subtypes in lungs at 0 and 3 d.p.i. with 200 or 2 × 10^5^ PFUs of PR8 (*n* = 5 mice/group). The presented data are representative of at least three (A, B) or two (C, D) independent experiments. Results are given as mean ± SD. Statistical significance was determined by two‐tailed Student's *t*‐test (A). ns, *p* > 0.05; ^**^ *p *< 0.01; ^****^ *p* < 0.0001.

To elucidate more precisely how fast γδ T cell respond to tracheal infection, we analyzed their total number during the first 3 d.p.i. We found that at 2 d.p.i., γδ T cell numbers had significantly increased with a peak of recruitment observed at 3 d.p.i. (Fig. 2A). Furthermore, we determined the different γδ T cell subtypes present in the trachea according to the expression of CCR6 and CD27. These markers enabled us to further classify them in IFN‐γ‐producing γδ T cells (CCR6^–^, CD27^+^) and IL‐17‐producing γδ T cells (γδ17 T cells; CCR6^+^, CD27^–^) 16, 25, 26 (Fig. 2B, left panels). The CCR6^+^ CD27^–^ γδ T cells showed a progressive increase in their frequency to 90% of total γδ T cells at 3 d.p.i. (Fig. 2B, upper right graph). Additionally, the absolute numbers of CCR6^+^ CD27^–^ γδ T cells were significantly higher at 2 and 3 d.p.i., indicating that they were the main subset contributing to the early γδ T cell infiltration (Fig. 2B, lower right graph). Subsequently, we analyzed the expression of Vγ1, Vγ4, and Vγ6 TCR chains by the tracheal γδ T cells. We found that the majority of the γδ T cells in the trachea were Vγ4^+^ in uninfected mice or at 3 d.p.i. (Fig. 2C). Furthermore, we observed that the frequency of Vγ4^+^ γδ T cell in the trachea was similar to that in the lungs and was not altered when mice were infected with a higher dose of PR8 (Fig. 2D).

### γδ T cells proliferate and get recruited to the trachea following influenza infection

**Figure 3:**
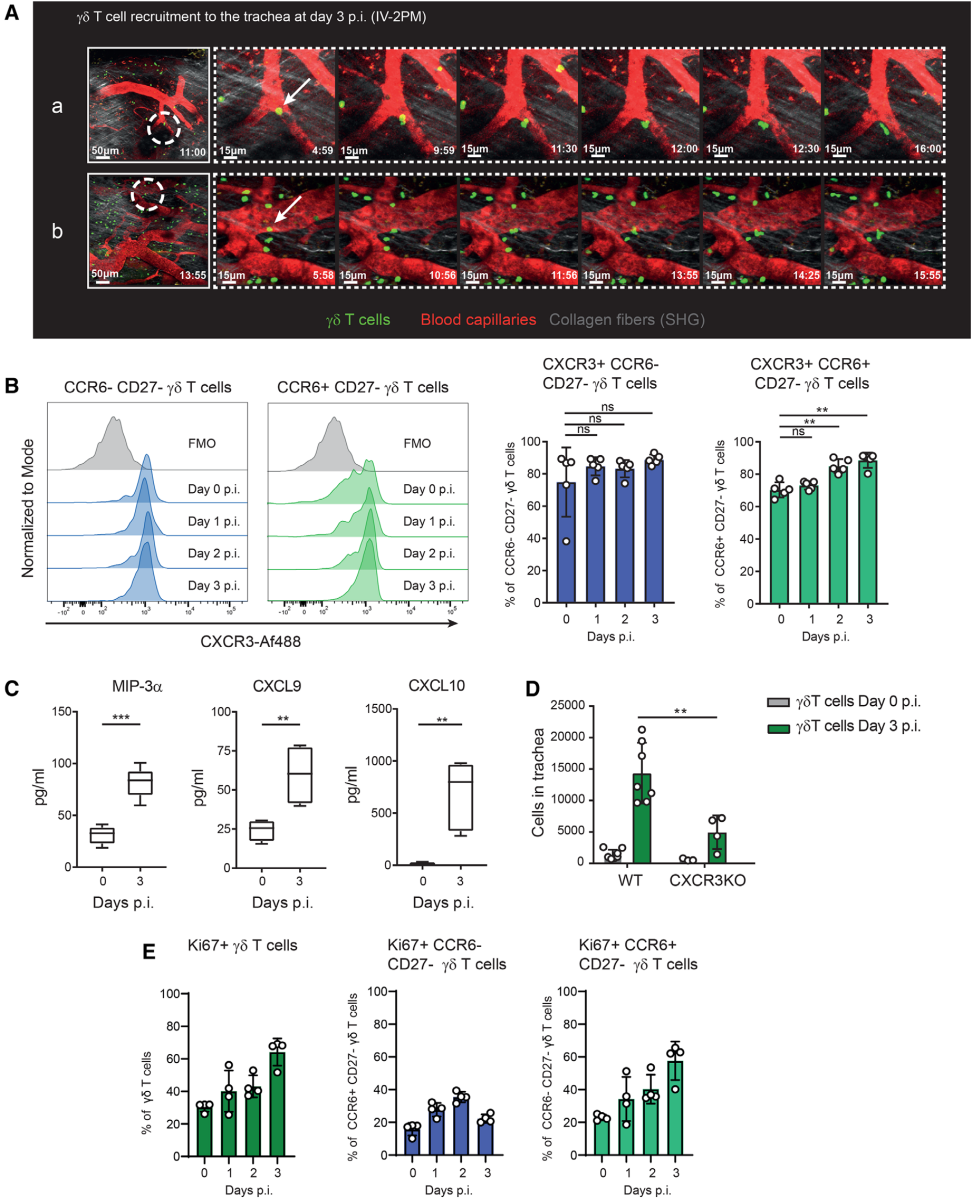
γδ T cells proliferate and get recruited to the trachea following influenza infection. (A) Sequential intravital 2‐photon micrographs (a,b) acquired at high magnification (40×) showing γδ T cells (green) extravasation into the tracheal tissue from blood vessels (red) at 3 d.p.i. (Supporting Information Movie 1). Second harmonic generation (SHG) signal from collagen is shown in grey. Dashed circles indicate magnified areas (dashed panels) and white arrows indicate cell extravasation. (B) (Left) Representative histograms showing the expression of CXCR3 in CCR6^–^ CD27^–^ γδ T cell and CCR6^+^ CD27^–^ γδ T cell and (right) quantification of the frequency of the CXCR3^+^ cells in the γδ T cell subtypes in trachea at 0, 1, 2, and 3 d.p.i. using flow cytometry (*n* = 5 mice/group). (C) Protein levels of secreted MIP‐3α, CXCL9, and CXCL10 in trachea at 0 and 3 d.p.i. determined by bead‐based immunoassay (LEGENDplex^TM^, BioLegend; *n* = 4–5 mice/group). (D) Flow cytometric quantification of γδ T cell in CXCR3KO mice at 3 d.p.i. (n = 3–7 mice/group). (E) Flow cytometric quantification of frequency of γδ T cell expressing Ki67 in trachea at 0, 1, 2, and 3 d.p.i. (*n* = 4 mice/group). The presented data are representative of at least three (B–D) or two (A, E) independent experiments. Results are given as mean ± SD. In (C), box plots show 25th to 75th percentiles and whiskers show minimum and maximum values. Statistical significance was determined by two‐tailed Student's *t*‐test. (ns, *p* > 0.05; ^**^ *p* < 0.01; ^***^ *p* < 0.001).

To study the recruitment of γδ T cells to the trachea, we performed intravital two‐photon microscopy in infected Tcrd‐H2BEGFP mice (Fig. 3A) in which γδ T cells expressed GFP 27. We could visualize the extravasation γδ T cells from blood capillaries to the tracheal tissue at 3 d.p.i. Following this, we investigated the expression of the chemokine receptor CXCR3 that is known to be expressed by γδ T cells and to participate in their recruitment during infection 28. The results showed that approximately 90% of the CCR6^–^CD27^–^ γδ T cells expressed CXCR3 during the first 3 d.p.i. However, 70% of CCR6^+^CD27^–^ γδ T cells expressed this receptor in uninfected animals and their frequency increased up to 90% at 3 d.p.i. (Fig. 3B). These results suggested different roles of CXCR3 in the recruitment of γδ T cell in the trachea. Therefore, to better examine the role of these receptors in the recruitment of γδ T cells, we determined the expression of their specific ligands (MIP‐3α and CXCL9/10, respectively) at 3 d.p.i. The analysis showed that the protein levels of the three chemokines were significantly higher at the time of γδ T cell recruitment (Fig. 3C). We further confirmed that the infiltration of γδ T cells was dependent on CXCR3, as CXCR3KO mice exhibited reduced numbers of this cell subset under the same conditions (Fig. 3D). However, a significant number of γδ T cells remained present in the trachea of infected CXCR3KO mice, which might indicate that γδ T cells could proliferate in the infected tissue. To investigate that, we analyzed the expression of the NF Ki67, which indicated that γδ T cells progressively increase their basal proliferating ratio (Fig. 3E). In addition, CCR6^+^CD27^–^ γδ T cells showed a higher proliferating ability compared to the CCR6^–^CD27^–^ subset.

### Vγ4^+^ γδ T cells produce IL‐17A during influenza infection

**Figure 4:**
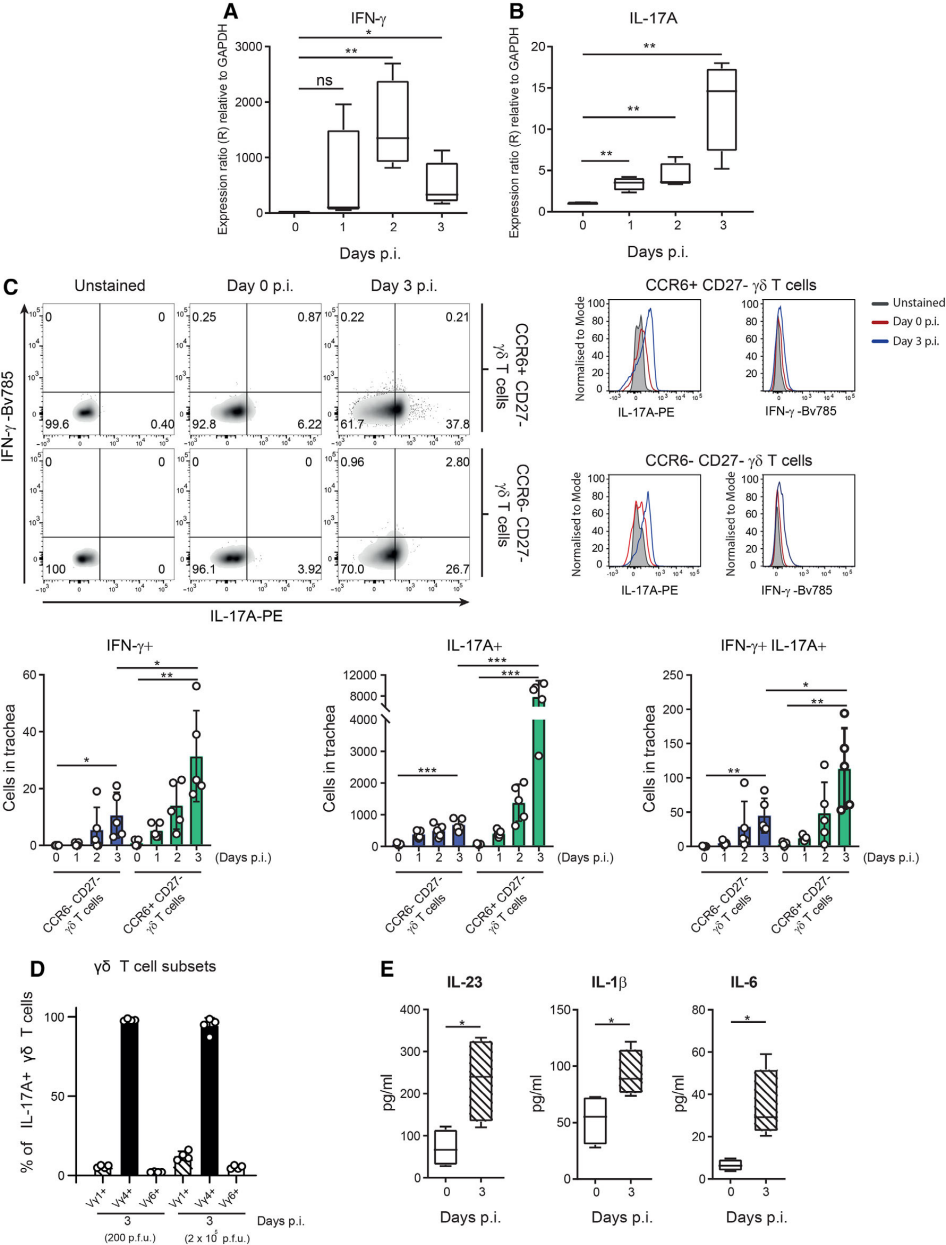
Vγ4^+^ γδ T cells produce IL‐17A during influenza infection. Time course showing RNA expression of IFN‐γ (A) and IL‐17A (B) in mouse trachea during the first 3 d.p.i. by qPCR (*n* = 4 mice/group). (C) Representative scatterplots and histograms showing the flow cytometric characterization of IFN‐γ‐ and/or IL‐17A‐producing cells from CCR6^+^ CD27^–^ γδ T cell and CCR6^–^ CD27 γδ T cell subsets in trachea at 3 d.p.i. (Upper panel) and their quantification (lower graphs; *n* = 4 mice/group). (D) Flow cytometric quantification of frequency of the different γδ T cell subtypes that express IL‐17A in trachea at 0 and 3 d.p.i. with 200 or 2 × 10^5^ PFUs of PR8 (*n* = 4 mice/group). (E) Protein levels of secreted IL‐23, IL‐1β, and IL‐6 in trachea at 0 and 3 d.p.i. determined by bead‐based immunoassay (LEGENDplex^TM^, BioLegend; n = 4 mice/group). The presented data are representative of at least three (A–C, E) or two (D) independent experiments. Results are given as mean ± SD. In (A), (B), and (E), box plots show 25th to 75th percentiles and whiskers show minimum and maximum values. Statistical significance was determined by two‐tailed Student's *t*‐test. ns, *p* > 0.05; ^*^ *p* < 0.05; ^**^ *p* < 0.01; ^***^ *p* < 0.001.

IFN‐γ and IL‐17A play an important role in the host defense against pathogens and are known to be expressed by γδ T cells early during infection 16. To characterize the role of γδ T cells during the early stages of the antiviral response against influenza virus, we assessed the expression of IFN‐γ and IL‐17A in the trachea. We observed a significant upregulation of the transcript levels of both IFN‐γ and IL‐17A during the first 2 d.p.i. (Fig. 4A and B, respectively). However, while the peak of expression of IFN‐γ was observed at 2 d.p.i., the expression of IL‐17A continued increasing until 3 d.p.i. Furthermore, we analyzed by intracellular staining the expression of both cytokines in the two main γδ T cell subsets and we observed that both subsets significantly expressed IL‐17A and almost no IFN‐γ after infection (Fig. 4C, upper panels). Moreover, we saw that the number of cells that expressed IFN‐γ, IL‐17A, or both cytokines together was significantly increased in both γδ T cell subsets at 3 d.p.i. (Fig. 4C, lower graphs). However, the number of cells expressing IFN‐γ or IFN‐γ and IL‐17A together represented <3% in both γδ T cell subsets. In addition, CCR6^+^CD27^–^ γδ T cells showed a higher frequency and total number of IL‐17A‐expressing cells at 3 d.p.i., compared to CCR6^–^CD27^–^ γδ T cells, indicating that the former is the main source of IL‐17A in the trachea postinfection (Fig. 4C). Next, we analyzed the Vγ TCR chain repertoire in IL‐17^+^ γδ T cells. We found that almost all the IL‐17A^+^ γδ T cells were Vγ4^+^ (Fig. 4D), which are known to express CCR6 and to lack expression of CD27 29. Furthermore, we observed that the frequency of Vγ4^+^ γδ T cell that express IL‐17A did not change after infection with a higher dose of influenza virus (Fig. 4D). We also measured in tracheal lavage the levels of the cytokines IL‐23, IL‐1β, and IL‐6 that are known to promote the expression of IL‐17A in γδ T cells 30. We observed a significant upregulation of all cytokines in comparison to uninfected controls (Fig. 4E), which coincided with the activation profile of γδ T cells at 3 d.p.i. (Fig. 1E).

### γδ T cells recruit neutrophils and NK cells and limit influenza infection in the trachea

**Figure 5:**
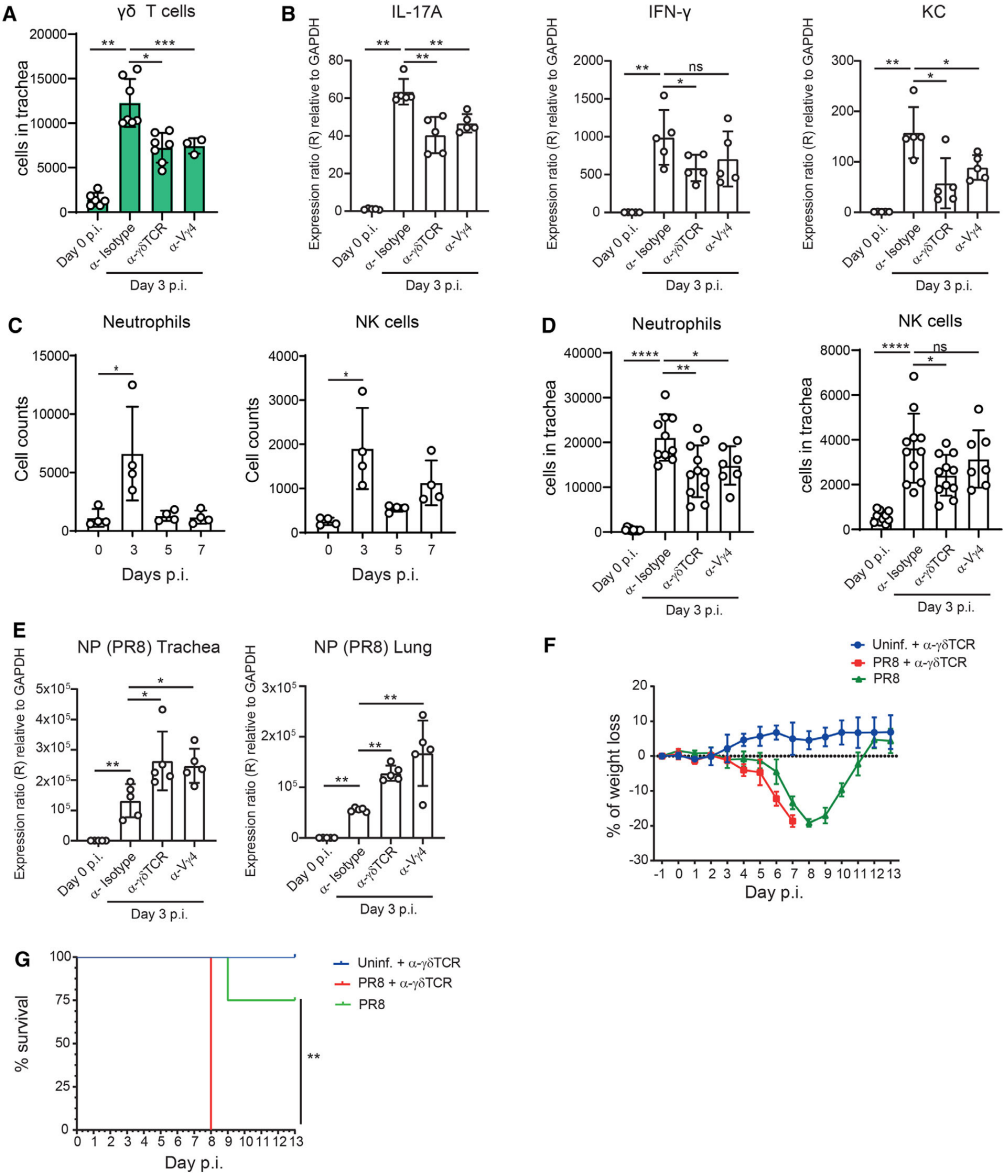
γδ T cells recruit neutrophils and NK cells and limit influenza infection in the trachea. (A) Flow cytometric quantification of total numbers of γδ T cell in trachea from mice treated with anti‐γδ TCR antibody (α‐γδ TCR), anti‐Vγ4 TCR antibody (α‐Vγ4), and with isotype control antibody (α‐isotype) at 3 d.p.i. (*n* = 3–7 mice/group). (B) RNA expression of IL‐17A, IFN‐γ, and CXCL1 in trachea from mice treated with anti‐γδ TCR antibody (α‐γδ TCR), anti‐Vγ4 TCR antibody (α‐Vγ4^+^), and with isotype control antibody (α‐Isotype) at 3 d.p.i. using qPCR (*n* = 5 mice/group). (C) Flow cytometric quantification total number of neutrophils and NK cells in trachea at 0, 3, 5, and 7 d.p.i. (*n* = 4 mice/group). (D) Flow cytometric quantification of total number of neutrophils and NK cells in trachea from mice treated with anti‐γδ TCR antibody (α‐γδ TCR), anti‐Vγ4 TCR antibody (α‐Vγ4^+^) and with isotype control antibody (α‐isotype) at 3 d.p.i. (*n* = 7–11 mice/group). (E) RNA expression of PR8 nucleoprotein (NP) in trachea and lungs from mice treated with anti‐γδ TCR antibody (α‐γδ TCR), anti‐Vγ4 TCR antibody (α‐Vγ4^+^), and with isotype control antibody (α‐isotype) at 3 d.p.i. using qPCR (*n* = 5 mice/group). Morbidity (F) and mortality (G) analysis of anti‐γδ TCR antibody‐treated and isotype control‐treated mice after infection with influenza virus (*n* = 4 mice/group). The presented data are representative of at least two (A, B, C, E, F, and G) independent experiments or two combined experiments (D). Results are given as mean ± SD. Statistical significance was determined by Mann–Whitney *U*‐test (A–E) or Mantel–Cox method (G). Results are given as mean ± SD. ns, *p* > 0.05; ^*^ *p* < 0.05; ^**^ *p* < 0.01; ^***^ *p *< 0.001; ^****^ *p* < 0.0001).

To study the role of γδ T cells, and specifically, Vγ4^+^ γδ T cells, during influenza infection, we deplete these cell populations in mice infected with influenza virus. Both treatments resulted in an approximately 50% reduction in the number of γδ T cells in the trachea at 3 d.p.i. (Fig. 5A; Supporting Information Fig. 1A). Then, we examined the impact of this reduction on the levels of IL‐17A and IFN‐γ. We observed that IL‐17A expression was significantly reduced in tracheas from animals treated with the anti‐γδ TCR or the anti‐Vγ4 TCR antibodies, while IFN‐γ levels were only affected by the treatment with the anti‐γδ TCR antibody (Fig. 5B; Supporting Information Fig. 1B). Furthermore, we observed a significant reduction in the levels of CXCL1 in both group of animals treated with anti‐γδ T or anti‐Vγ4^+^ γδ T cells depleting antibodies (Fig. 5B). CXCL1 is known to be one of the major chemoattractants of neutrophils to the infected sites 31. It is also known that IL‐17A derived from γδ T cells promotes the recruitment of neutrophils 32, 33. To examine the relationship between γδ T cells and neutrophils in the context of influenza infection, we analyzed the number of neutrophils in the trachea during the first 7 d.p.i. We observed that their number increased significantly at 3 d.p.i. (Fig. 5C; Supporting Information Fig. 1C). Moreover, we observed that the number of neutrophils was reduced in the groups treated with depleting antibodies (Fig. 5D).

In addition, we observed that NK cells were recruited to the infected trachea at 3 d.p.i. (Fig. 5C, Supporting Information Fig. 1C). However, NK cells numbers were significantly reduced only in animals treated with the anti‐γδ TCR antibody (Fig. 5D). We also confirmed that other T cell subsets, dendritic cells (DC) or macrophages were not affected by the treatment with anti‐γδ TCR antibody (Supporting Information Fig. 1D). Finally, the analysis of neutrophil numbers in tracheas from infected IL‐17A/IL‐17F double KO (IL‐17AFKO) mice, confirmed the role of IL‐17 in neutrophil recruitment during influenza infection (Supporting Information Fig. 1E). To investigate how the reduction in the number of γδ T cells affected the progression of influenza infection, we measured the expression of influenza virus‐related genes and viral titers in trachea and lung at 3 d.p.i. (Fig 5E; Supporting Information Fig. 1F). We detected that the expression of influenza‐related genes were higher in mice treated with anti‐γδ TCR and anti‐Vγ4 TCR antibodies, compared to WT control (Fig. 5E). These results also correlated with increased weight loss (Fig. 5F) and mortality (Fig. 5G) during infection of anti‐γδ TCR antibody‐treated mice, in comparison to the control group.

## Discussion

Previous studies have shown that γδ T cells recruited to the lungs at later time points (10 and 15 days) after influenza infection ameliorated the recovery of the animals 12 and protected them from a secondary influenza infection 13. However, our results indicated that γδ T cells are already present in high numbers in the trachea at 3 d.p.i. and their frequency is greater than conventional CD4^+^ and CD8^+^ αβ T cells. This is in accordance with previous studies that examined the lungs of mice in which γδ T cells were also found to be recruited at early time points after influenza infection 24. In addition, our results indicated that early‐recruited γδ T cells were highly activated, as assessed on the basis of CD69 expression. Whether this activation was a result of direct recognition of viral particles is not known. However, it has been shown that γδ T cells express CD69 after recognition of influenza‐infected cells 34. Thus, our data suggest that γδ T cells are early sensors during influenza infection in the trachea, whereas αβ T cells exert their function at later time points after priming by APCs.

Murine γδ T cells consist of various subsets characterized by their distinct anatomical locations and their commitment to the production of either IFN‐γ or IL‐17 35. In the case of IL‐17, production is restricted mostly to Vγ4^+^ and Vγ6^+^ γδ T cells, while Vγ1^+^ γδ T cells are known to mainly produce IFN‐γ 29. IL‐17‐ and IFN‐γ‐producing γδ T cells can be broadly characterized on the basis of the expression of CCR6 and CD27, respectively 16,25. Our results demonstrated that the majority of the infiltrating γδ T cells were CCR6^+^ CD27^–^ and expressed the Vγ4 TCR chain, which coincided with the phenotype of IL‐17‐producing γδ T cells (γδ17 T cells). Accordingly, another study showed that Vγ4^+^ γδ T cells were recruited in the lungs at early times postinfection 24.

Interestingly, we observed that the increase of γδ T cells in the trachea during infection was due to two events: their ability to proliferate in situ, and their active recruitment from the blood. Regarding γδ T cell migration, we saw that a high percentage of the CCR6^+^ cells also expressed the chemokine receptor CXCR3. This was in accordance with a previous study in which it was found that CXCR3 can be co‐expressed with CCR6 in murine γδ17 T cells 36. Furthermore, we demonstrated that specific ligands for both CCR6 and CXCR3 (MIP‐3α and CXCL9/10, respectively) were expressed in the trachea after infection, suggesting their involvement in the recruitment of γδ T cells.

Using the same infection model, we previously showed that inflammatory DCs (IDCs) were the major producers of CXCL9 and CXCL10 at 3 d.p.i. 2. Moreover, earlier studies have shown a critical role of CXCR3 in the recruitment of γδ T cells in influenza‐infected sites, which facilitated the elimination of pathogens 28. Taking this into account, we hypothesize that IDC are involved in the infiltration of γδ T cell. In addition, we observed a minor population of CCR6^–^CD27^–^ γδ T cells, which does not fully correspond to any of the above‐mentioned γδ T cell subsets. This cell population was present in the trachea at steady state and the frequency of those cells, among the total γδ T cells, decreased over time. Although a full characterization of this subset is required, previous reports have indicated that CCR6 drives the migration of γδ17 T cells to the inflamed tissue but its expression is lost after activation to prevent the accumulation of γδ T cells in uninflamed tissues 37. We showed that, in addition to CCR6^+^ CD27^–^ γδ T cells, the numbers of CCR6^–^CD27^–^ γδ T cells increased along with their CXCR3 and IL‐17A expression during infection. Therefore, it is possible that this population represents a different maturation state of the recruited CCR6^+^CD27^–^ γδ T cells.

γδ T cells are known to rapidly secrete IL‐17A and IFN‐γ much earlier than adaptive αβ T cells 38. IL‐17A and IFN‐γ are inflammatory cytokines that promote the recruitment of inflammatory cells 30 and the generation of an antiviral state 17, respectively. In our study, we evaluated the ability of the different γδ T cell subsets to produce these two cytokines at early time points. We showed that IL‐17A in the trachea was mainly produced by Vγ4^+^ γδ T cells during the innate immune response to influenza infection, which was in agreement with previous studies that focused on the lungs 24. For γδ T cells to produce IL‐17A in a TCR‐independent manner, the presence of IL‐23, IL‐1β, and IL‐6 is necessary 30. In this study, we reported an early production of these cytokines in the trachea postinfection, which suggested that γδ T cells are activated in a cytokine‐dependent manner during influenza infection. Surprisingly, we observed that a small percentage of γδ T cells was able to produce only IFN‐γ or both IL‐17A and IFN‐γ. Although γδ17 T cells are known to be fully committed cells to the production of IL‐17 35, it has been shown that stimulation with IL‐1β and IL‐23 can also induce the co‐production of IFN‐γ and IL‐17A 39. Moreover, we demonstrated that the reduction of the number of γδ T cells using a depleting antibody affected the expression of both cytokines. This data suggested that γδ T cells are major producers of IL‐17A at early time points. However, in the case of IFN‐γ, our results indicated that γδ T cell reduction also affected the recruitment of NK cells that are able to produce large amounts of IFN‐γ 40. In a recent study 2, we demonstrated that NK cell recruitment to the trachea during influenza infection was dependent on the expression of cytokines produced by the IDC. Moreover, It is also known that γδ T cell are able to deliver activating signals to DC and monocytes 29. Therefore, we hypothesize that γδ T cells may be able to deliver activating signals to IDC during infection, which would support the observed recruitment of NK cells and the production of IFN‐γ.

We observed that the early production of IL‐17A by Vγ4^+^ γδ T cells modulates neutrophil recruitment during influenza infection in the trachea. Previous studies have shown that IL‐17A signals through the IL‐17 receptor A (IL‐17RA) to upregulate the production of chemokines such as CXCL1, which in turn regulates neutrophil migration to mucosal sites 32. Accordingly, we showed that the treatment with antibodies that generally deplete γδ T cells or antibodies that only deplete Vγ4^+^ γδ T cells reduced IL‐17A levels, which correlated with lower levels of CXCL1 and neutrophil recruitment at 3 d.p.i. Moreover, neutrophils are known to play a protective role during influenza infection by limiting influenza virus replication during the early and later phases of infection 41. In agreement to this, we demonstrated that a reduction in the number of γδ T cells or Vγ4^+^ γδ T cells at early stages postinfection correlated with a decrease in the number of neutrophils at the site of infection and an increase of the viral load in the trachea and lungs. Although neutrophils seem to be involved in the control of viral spread, we cannot discard the possibility that γδ T cells may perform additional functions that limit virus dissemination. More specifically, in the context of influenza infection, human Vγ9Vδ2 T cells have been shown to contribute to the control of viral infection in vitro 14 and in humanized mice 15 by direct killing of influenza‐infected cells. Furthermore, the use of the anti‐γδ TCR antibody also affected the recruitment of NK cells, which are critical for viral clearance in the respiratory tract 2. Therefore, the increased susceptibility shown by animals treated with the anti‐γδ TCR antibody might also be related to this effect. However, our study indicated that the reduction of Vγ4^+^ γδ T cell does not lead to an impaired NK cell recruitment or IFN‐γ production during infection. Thus, the increased viral levels shown in animals treated with anti‐Vγ4 TCR antibodies can solely be associated with the reduced number of neutrophils. Whether another subtype of γδ T cells, such as Vγ1^+^ γδ T cells might be specifically associated with the infiltration of NK cells needs to be further evaluated.

Our study highlights, for the first time, the importance of the γδ17 T cells during the early stages of influenza infection. However, a previous study has shown a detrimental role of these cells in acute lung infection models 24. We speculate that the observed differences might be related to the infection model. While in our work mice were infected with a low volume and dose of virus to mimic the natural model of infection, Xue et al. 24 employed a high dose of the virus, which generated a severe infection in the lungs. We demonstrated that the generation of an early acute pulmonary infection led to an increased frequency of γδ T cells in the lungs and a reduction in their numbers and activation in the trachea. This might explain why in the report by Xue and colleagues an augmented presence of γδ T cells in the lungs is detrimental for the survival of infected mice.

In conclusion, we demonstrated that the early recruitment of the Vγ4^+^ γδ T cell subset is an important event of the initial immune reaction against influenza infection that allows the rapid secretion of IL‐17A and, thus, contributes to the recruitment of protective neutrophils in the tracheal mucosa. Therefore, the immune response generated by γδ T cells contributes to the early elimination of virus‐infected cells in the trachea. These findings suggest the potential use of the IL‐23/IL‐17 axis in a therapeutic approach for the enhancement of the function of γδ17 T cells against multiple strains of influenza virus.

## Methods

### Mice

C57BL/6 mice, CXCR3KO 42, IL‐17AFKO 43, and Tcrd‐H2BEGFP 27 were bred in‐house or acquired from Janvier labs. Mice were maintained in specific pathogen‐free facilities at the Institute for Research in Biomedicine, Bellinzona. Experiments were performed in accordance with the Swiss Federal Veterinary Office guidelines and animal protocols were approved by the local veterinarian authorities.

### Antibodies

The fluorescently labeled antibodies for cell surface and intracellular staining are listed in Supporting Information Table 1. Additionally, anti‐mouse TCR γ/δ antibody (UC7‐13D5, BioXcell), anti‐mouse TCR Vγ4 (2.11, BioXcell), and polyclonal Armenian hamster IgG antibody (BioXcell) as isotype control were used for γδ T cell depletion.

### Influenza virus production, infection and survival assay

The influenza virus strain A/PR/8/34 was grown, purified, inactivated, and labeled as described previously 44. Age‐matched (6‐ to 8‐wk‐old) female mice were anesthetized with a mix of ketamine (100 mg/kg bodyweight, Parke Davis) and xylazine (10 mg/kg bodyweight, Bayer) and intranasally inoculated with 40 µL (20 µL on each nare) containing 200 or 2 × 10^5^ PFUs of influenza virus. In survival studies, mice were monitored daily for up to 12 days and sacrificed when weight loss was superior to 20 %. For γδ T cell depletion 400 µg of anti‐mouse TCR γ/δ antibody, the anti‐mouse TCR Vγ4 or the corresponding isotype control was administered intraperitoneally one day before infection, at 1 and at 3 d.p.i.

### Flow cytometry

Flow cytometry analysis was conducted accordingly to the recently published guidelines 45. Organs were mechanically disrupted with scissors and digested for 45 min at 37°C in an enzyme mix composed of: DNase I (0.28 mg/mL, Amresco, Fountain Parkway Solon, OH), and 0.26 units/mL of Liberase TL Research Grade (Roche, Basel, Switzerland)) in RPMI 1640 Medium (Gibco, Bleiswijk, Netherlands) followed by a stop solution of 2 mM EDTA (Sigma–Aldrich, San Luis, MO) and 2% heat‐inactivated filter‐sterilized FCS (Thermo Fisher Scientific, Waltham, MA) in PBS (Sigma–Aldrich, San Luis, MO). Single cell populations were obtained by forcing the remaining tissue pieces through a 40‐µm strainer followed by lysis of RBCs. Fc receptors from the isolated cells were blocked (αCD16/32, Biolegend, San Diego, CA) followed by surface staining and analysis by flow cytometry on a LSRFortessa^TM^ (BD Biosciences, Franklin Lakes, NJ). Where indicated, intracellular staining was performed according to eBioscience™ Intracellular Fixation & Permeabilization Buffer Set (eBioscience, Santa Clara, CA) following the manufacturer's instructions. Dead cells were excluded using ZombieAcqua fixable viability dye (Biolegend, San Diego, CA) and data were analyzed using FlowJo software (TriStar Inc, Phoenix, AZ). For detection of Vγ6^+^ cells, samples were pre‐stained with GL3 followed by 17D1.

### Cytoplex assay

LEGENDPlex^TM^ assays (Mouse Proinflammatory Chemokine Panel and Mouse Inflammation Panel; Biolegend, San Diego, CA) were performed to monitor cytokine/chemokine expression. Briefly, tracheas were collected and the luminal side was washed five times with 100 µL of ice‐cold PBS. Tracheal washes were centrifuged at 1500 rpm for 5 min and the supernatant was collected. Twenty‐five microliters of supernatant were processed following the manufacturer's instructions. Samples were analyzed by flow cytometry on LSRFortessa^TM^ (BD Biosciences, Franklin Lakes, NJ) and data were analyzed using LEGENDPlex^TM^ software (Biolegend, San Diego, CA).

### Real‐time quantitative PCR

To measure the expression levels of the cytokines IFN‐γ, IL‐17A, KC, and NP (PR8), the following sets of primers were designed (direction 5’‐3’): IFN‐γ FW:GAGGAACTGGCAAAAGGATG; RV:GCTGATGGCCTGATTGTCTT‐3’; IL‐17A FW:AGCTGGACCACCACATGAAT and RV:ACACCCACCAGCATCTTCTC; CXCL1 FW:TCAGGGGCTGGAATAAAA and RV:ACAGGTGCCATCAGAGCAGT; NP (PR8) FW:TGCCTGCCTGTGTGTATGG and RV:AGGCTGTACACTTGGCTGTTT. Tracheas and the upper right lobe of the lungs were collected at the specified time point postinfection in 700 µL of TRIzol™ Reagent (ThermoFisher Scientific, Waltham, MA), disrupted in lysing matrix D 1.4 mm ceramic sphere tubes using FastPrep^®^‐24 tissue disruption (MP Biomedicals, Illkirch‐Graffenstaden, France) and RNA was isolated using an RNAeasy Mini kit (Qiagen, Hilden, Germany). One microgram of cDNA was synthesized using a cDNA synthesis kit (Applied Biosystems, Foster City, CA) following the manufacturer's recommendations. For the RQ‐PCR reaction, a SYBR® Master Mix (Applied Biosystems, Foster City, CA) was used and samples were run on a 7900HT Fast Real‐Time PCR System (Applied Biosystems, Foster City, CA). Cytokine mRNA levels were expressed relative to GAPDH expression (Primers: GAPDH FW:ACATCATCCCTGCATCCACT and RV:AGATCCACGACGGACACATT). The Pfaffl method 46 was used to calculate the relative expression of the transcripts.

### Viral Titers

Influenza titres from trachea homogenates were measured by a 50% tissue culture infective dose (TCID_50_) assay. Briefly, tracheas were aseptically removed from mice, weighed, and disrupted in 1 mL of ice‐cold sterile PBS. The determination of TCID_50_ was carried out using 96‐well plates containing confluent Madine‐Darby canine kidney (MDCK) cell monolayers. The MDCK cells were incubated with serial threefold dilutions of influenza virus culture supernatant in infection medium for 1 h at 37°C. After, the monolayer was rinsed with PBS, overlaid with infection medium, and incubated at 37°C for 4 days. To identify influenza virus‐positive wells, the monolayers were stained with Crystal Violet (Sigma–Aldrich, San Luis, MO) in 70% methanol. Titers were expressed as the dilution of trachea extract at which 50% of the MDCK cultures revealed virus growth, as calculated by the Spearman and Karber method.

### Intravital two‐photon microscopy and analysis

Intravital microscopy of γδ T cell recruitment to the trachea was perform in Tcrd‐H2BEGFP at 3 d.p.i. as previously described 47, 48. Deep tissue imaging was performed on a customized two‐photon platform (TrimScope, LaVision BioTec, Bielefeld, Germany). Two‐photon probe excitation and tissue second‐harmonic generation (SHG) were obtained with a set of two tunable Ti:sapphire lasers (Chamaleon Ultra I, Chamaleon Ultra II, Coherent, Santa Clara, CA) and an optical parametric oscillator that emits in the range of 1010–1340 nm (Cha‐ maleon Compact OPO, Coherent, Santa Clara, CA), with output wavelength in the range of 690–1080 nm. For the in vivo analysis of cell movement, two‐photon micrographs were acquired in full Z stacks of 40 µm every 30 s. To visualize γδ T cell, Imaris 9.3.1 software (Bitplane, Belfast, UK) was used.

### Statistics

All data are expressed as the mean ± SD. For statistical analyses and data presentation Prism 7 (GraphPad Software, GraphPad Software Inc, San Diego, CA) was used. The Shapiro–Wilk test was used to assess whether data followed normal distribution. Accordingly, group comparisons were assessed using two‐tailed Student's or Mann–Whitney *U*‐test. For statistical analysis between survival curves Mantel–Cox method was used. Statistical significance was defined as: ns, *p *> 0.05; ^*^ *p* < 0.05; ^**^ *p* < 0.01; ^***^ *p *< 0.001; ^****^ *p *< 0.0001.

## Authors Contributions

M.P.‐S. and S.F.G. conceived the project, designed experiments, analyzed, and interpreted the results. M.P.‐S. performed all the experiments. I.L. helped with experiments. Y.F. advised on the experiments, interpreted results, and helped to develop protocols. S.F.G. and M.P.‐S. wrote the manuscript with the help of Y.F. S.F.G. directed the study.

## Conflict of interest

The authors declare no commercial or financial conflict of interests.

**Abbreviations**
DN: double negative
d.p.i.: days postinfection
IDC: inflammatory DC

## Supporting information

Supporting Information

Supporting Information
