# Supplementary material for: Early production of IL‐17A by γδ T cells in the trachea promotes viral clearance during influenza infection in mice
Source: Eur J Immunol. 2019 Dec 4;50(1):97–109. doi: 10.1002/eji.201948157 (PMC7003741; doi:10.1002/eji.201948157)
Supplement: Supplementary file 1 — Supporting Information [file EJI-50-97-s001.pdf]

European Journal of Immunology

Supporting Information
for
DOI 10.1002/eji.201948157

Miguel Palomino-Segura, Irene Latino, Yagmur Farsakoglu
and Santiago F. Gonzalez

Early production of IL-17A by $\gamma\delta$ T cells in the trachea promotes viral clearance during influenza infection in mice

© 2019 The Authors. *European Journal of Immunology* published by WILEY-VCH Verlag GmbH & Co. KGaA, Weinheim.

Early production of IL-17A by Vγ4+ γδ T cells in the trachea promotes neutrophil recruitment and viral clearance during influenza infection.

Miguel Palomino-Segura[1,2], Irene Latino[1], Yagmur Farsakoglu[1,3] and Santiago F. Gonzalez[1*]

Supplementary information

Supplementary Figure 1

Supplementary Movie 1

Supplementary Table 1

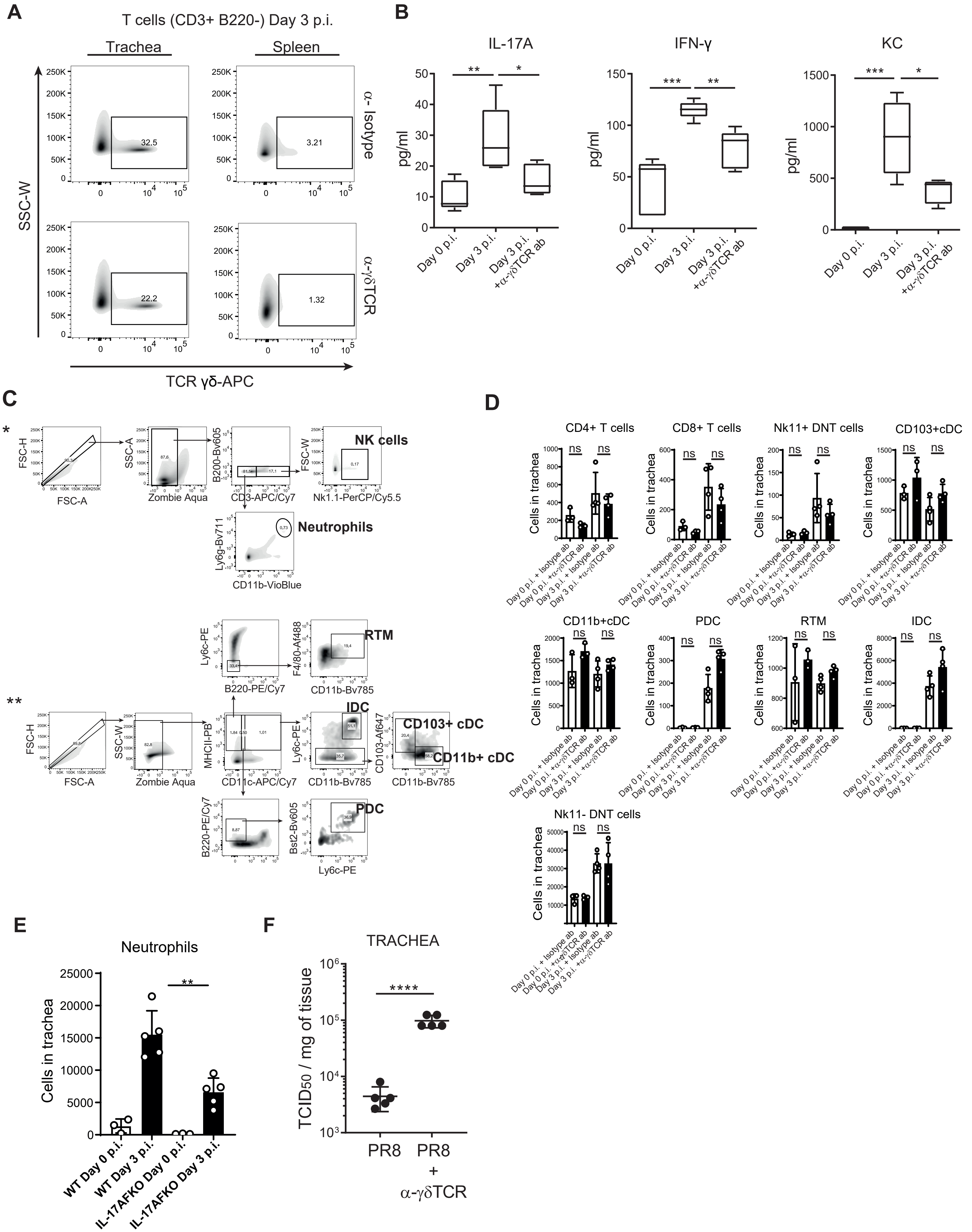

A
T cells (CD3+ B220-) Day 3 p.i.
Trachea
Spleen
α- Isotype
α-γδTCR
SSC-W
TCR γδ-APC
32.5
3.21
22.2
1.32
B
IL-17A
IFN-γ
KC
pg/ml
Day 0 p.i.
Day 3 p.i.
Day 3 p.i. +α-γδTCR ab
C
NK cells
Neutrophils
FSC-H
FSC-A
SSC-A
Zombie Aqua
B200-Bv605
CD3-APC/Cy7
FSC-W
Nk1.1-PerCP/Cy5.5
Ly6g-Bv711
CD11b-VioBlue
RTM
Ly6c-PE
B220-PE/Cy7
F4/80-Af488
CD11b-Bv785
IDC
CD103+ cDC
CD11b+ cDC
SSC-W
MHCII-PB
CD11c-APC/Cy7
CD103-Af647
PDC
Bst2-Bv605
D
CD4+ T cells
CD8+ T cells
Nk11+ DNT cells
CD103+cDC
CD11b+cDC
PDC
RTM
IDC
Nk11- DNT cells
Cells in trachea
ns
Day 0 p.i. + Isotype ab
Day 0 p.i. +α-γδTCR ab
Day 3 p.i. + Isotype ab
Day 3 p.i. +α-γδTCR ab
E
Neutrophils
Cells in trachea
WT Day 0 p.i.
WT Day 3 p.i.
IL-17AFKO Day 0 p.i.
IL-17AFKO Day 3 p.i.
F
TRACHEA
TCID50 / mg of tissue
PR8
PR8 + α-γδTCR

Supplementary Figure 1. *(**A**) Representative scatterplots showing the reduction of γδ T cell in trachea and spleen from mice treated with anti-γδ TCR antibody (α-γδ TCR) at day 3 p.i.. (**B**) Levels of secreted IL-17A, IFN-γ and KC in trachea from mice treated with anti-γδ TCR antibody (α-γδ TCR ab) and with isotype control antibody (day 3 p.i.) at day 3 p.i. (n = 4-5 mice/group). (**C**) (Upper panels (*)) flow cytometric characterisation of neutrophils and NK cells, and (lower panels (**)) inflammatory dendritic cells (IDC), plasmacytoid DC (PDC), CD103+ conventional DC (CD103+ cDC), CD11b+ conventional DC (CD11b+ cDC) and resident respiratory tract macrophages (RTM) in mouse trachea according to* [6]*. (**D**) Flow cytometric quantification of total numbers of T cell subsets, DC subsets and macrophages in trachea from mice treated with anti-γδ TCR antibody (α-γδ TCR ab) and with isotype control antibody (α-Isotype ab) at day 0 and 3 p.i. (n = 3-4 mice/group). (**E**) Flow cytometric quantification of γδ T cell in IL-17AFKO mice at day 3 p.i. (n= 3-5 mice/group). (**F**) Virus titres in trachea determined by $TCID_{50}$ from mice infected with influenza virus and treated with an anti-γδ TCR antibody (PR8+ α-γδ TCR) or with the isotype control antibody at day 3 p.i. (n = 5 mice/group). The presented data are representative of at least two (A, D, E and F) or three (B) independent experiments. Results are given as mean ± SD. In B box plots show 25th to 75th percentiles and whiskers show minimum and maximum values. Statistical significance was determined by Two-tailed Student's t-test (B, F) or Mann-Whitney U-test (D, E). (ns $p>0.05$; *$p<0.05$; **$p<0.01$; ***$p<0.001$; ****$p<0.0001$).*

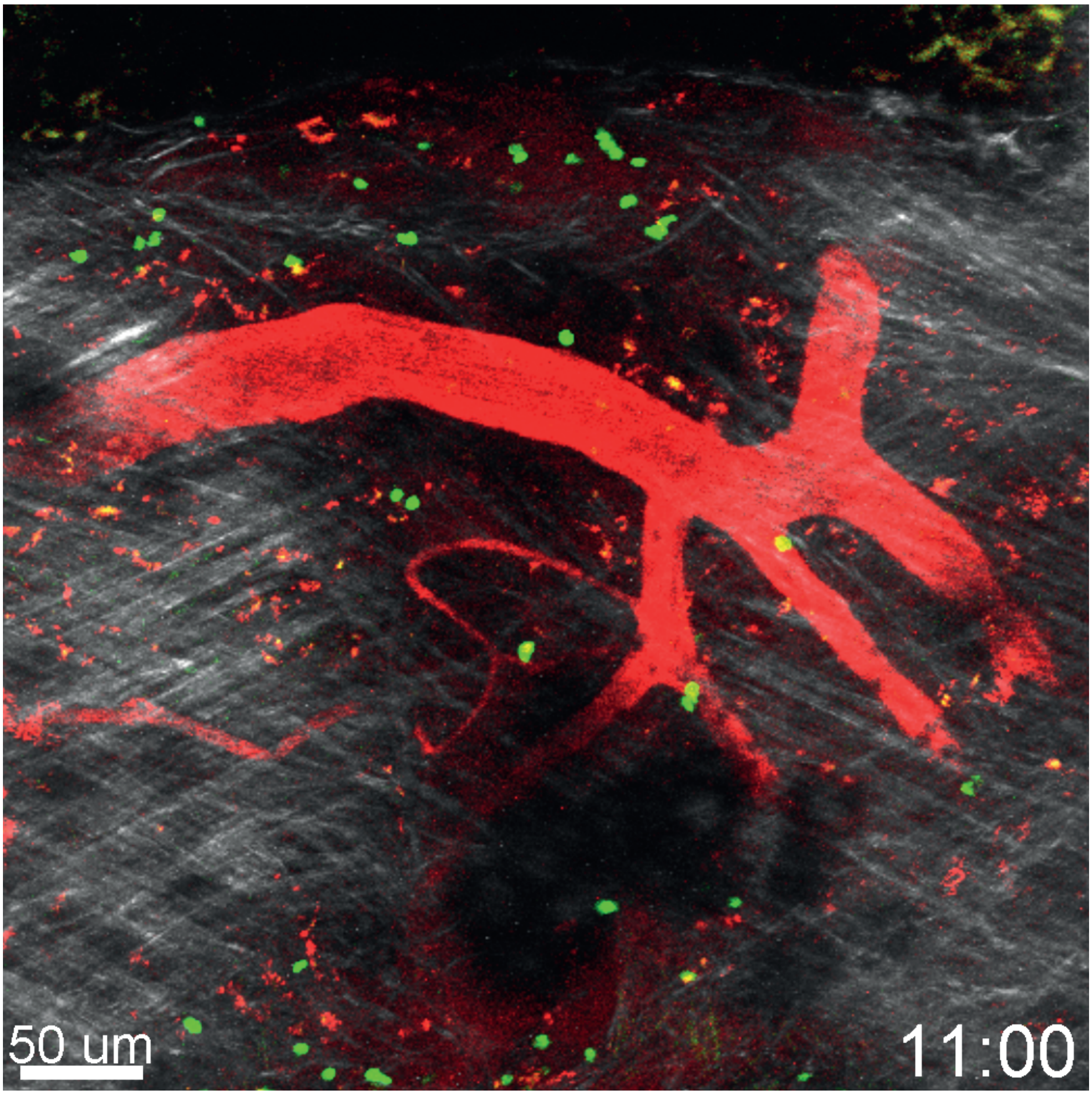

50 um
11:00

Supplementary Movie 1. **In vivo *imaging of γδ T cell recruitment to the trachea after influenza infection.*** *(First part) Two videos (a,b) of 30-45 min time-lapse 3D image showing the dynamics of γδ T cells (green, small and motile), SHG signal from fibrillary collagen (grey) and blood vessels (red) in trachea at day 3 p.i. (Second part) magnified representative sequences showing γδ T cell extravasation from the blood vessels into the tracheal tissue. Scale bar represents 50 μm.*

Supplementary Table 1.

**Antibody target**	**Clone**	**Color**	**Manufacturer**
B220	RA3-6B2	PE/Cy7, BV605	Biolegend
CD11b	M1/70	AF488, BV785,	Biolegend
CD45	30-F11	AF700	Biolegend
CD3ε	eBio500A2	eFluor450	eBioscience
NK1.1	PK136	AF488, PerCP-Cy5.5	Biolegend, eBioscience
CXCR3	CXCR3-173	AF488	Biolegend
CD69	H1.2F3	FITC, PE	Biolegend
IFN-γ	XMG1.2	BV785	Biolegend
IL17-A	TC11-18H10.1	PE	Biolegend
Ly-6G	1A8	BV711	Biolegend
CD16/32	93	Pure	Biolegend
CD8α	53-6.7	PE/Cy7, BV650	Biolegend
CD4	GK1.5	APC/Cy7	Biolegend
γδTCR	GL3	APC	Biolegend
CD27	LG.7F9	PE/Cy7	eBioscience
CCR6	29-2L17	PE/Dazzle 594	Biolegend
αLy-6C	HK1.4	PE	Biolegend
αF4/80	BM8	AF488	Biolegend
αCD103	2E7	AF647	Biolegend
αCD11b	M1/70	BV785, VioBlue	Biolegend, Miltenyi
αI-A/I-E (MHCII)	M5/114.15.2	Pacific Blue	Biolegend
αCD11c	N418	APC/Cy7	Biolegend
Bst2	927	Bv605	Biolegend
TCR Vγ4 (2)	UC3-10A6	FITC, Pure	Biolegend, BioXcell
TCR Vγ1.1/Cr4	2.11	PerCP/Cy5.5	Biolegend
TCR Vγ6	17D1	DyLight 800*	donated by Dr. Immo Prinz (U. of Hannover)
TCR γ/δ	UC7-13D5	Pure	Bioxcell
Armenian hamster IgG	(isotype control)	Pure	Bioxcell

**In-house labelled with Lightning-Link® Rapid DyLight® 800 conjugation kit (Innova Biosciences).*
